# Cutaneous neurofibromas in Neurofibromatosis type I: a quantitative natural history study

**DOI:** 10.1186/s13023-018-0772-z

**Published:** 2018-02-07

**Authors:** Ashley Cannon, Mei-Jan Chen, Peng Li, Kevin P. Boyd, Amy Theos, David T. Redden, Bruce Korf

**Affiliations:** 1 Department of Genetics, University of Alabama, 720 20th Street South, Birmingham, AL 35294 USA; 2 Department of Biostatistics, University of Alabama, Birmingham, AL USA; 3 Department of Dermatology, University of Alabama, Birmingham, AL USA

**Keywords:** Neurofibromatosis type 1, Cutaneous neurofibromas, Natural history study, Random effect modeling

## Abstract

**Background:**

Neurofibromatosis type 1 (NF1) is a genetic disorder characterized by a predisposition to develop multiple benign tumors. A major feature of NF1 is the development of localized cutaneous neurofibromas. Cutaneous neurofibromas manifest in > 99% of adults with NF1 and are responsible for major negative effects on quality of life. Previous reports have correlated increased burden of cutaneous neurofibromas with age and pregnancy, but longitudinal data are not available to establish a quantitative natural history of these lesions. The purpose of this study is to conduct a prospective natural history study of 22 adults with NF1 over an 8-year period to quantify cutaneous neurofibroma number and size.

**Results:**

The average monthly increase in volume for cutaneous neurofibromas was 0.37 mm^3^ in the back region (95% CI (0.23, 0.51), *p* < 0.0001), 0.28 mm^3^ in the abdominal region (95% CI (0.16, 0.41), *p* < 0.0001), and 0.21 mm^3^ in the arm/leg region (95% CI (0.08, 0.34), *p* = 0.0022). The number of cutaneous neurofibromas significantly increased in the back (slope = 0.032, *p* = 0.011) and abdominal (slope = 0.018, *p* = 0.026) regions, while the leg/arm regions retained a positive trend (slope = 0.004, *p* = 0.055).

**Conclusions:**

The number and volume of cutaneous neurofibromas significantly increased over an 8-year timespan; however, the rate of increase is variable by individual and body region. These findings may provide insight into cutaneous neurofibroma development and benefit researchers considering clinical trials targeting cutaneous neurofibromas.

**Electronic supplementary material:**

The online version of this article (10.1186/s13023-018-0772-z) contains supplementary material, which is available to authorized users.

## Background

Neurofibromatosis type 1 (NF1) is an autosomal dominant disorder affecting approximately 1:3000 individuals worldwide and is characterized by cutaneous, neurological, skeletal, and neoplastic manifestations. Localized cutaneous neurofibromas (cNFs) derive from peripheral nerves and manifest within the dermis and subcutaneous tissue. Although these lesions are not life threatening, they can lead to significant morbidity. A population-based survey in Wales found cNFs in > 99% of adults with NF1 [1], making this the most common tumor manifestation in adults. The current scientific literature of survey-based and cross-sectional studies collectively indicate that cNFs usually appear around puberty, increase with age, and undergo periods of rapid growth in puberty and pregnancy [1–3]. None of these studies obtained longitudinal data to establish a quantitative natural history of these lesions.

Visibility of cNFs are associated with major negative effects on quality of life for individuals with NF1 [4–6]. Treatment of cNFs is limited to surgical excision or destruction (e.g. laser or electrodessication) [7–14]. Removing all lesions is not usually feasible due to the abundance of cNFs, a scar will result at each site, and tumors can regenerate from remaining cells. The development of non-surgical treatment and clinical trials directed at cutaneous neurofibromas has been hampered by the difficulty in measuring and counting cNFs and lack of detailed information on the natural history of the lesions.

The present study has expanded on a previously described approach to quantify the number of cNFs [15] and to measure cNF size. This approach allowed us to elicit quantitative data on the rate of appearance and growth of cutaneous neurofibromas from a prospective natural history study of cutaneous neurofibromas in adult NF1 patients over 8 years. This dataset establishes both a method for quantitative assessment of cutaneous neurofibromas and a baseline dataset on the progression of untreated tumors that will pave the way for clinical trials of medical treatment for these tumors.

## Methods

### Participants

All participants were evaluated at the University of Alabama at Birmingham, a regional medical center with the only neurofibromatosis clinic in the state. Inclusion criteria included adult patients (≥18 years) who met clinical diagnostic criteria for NF1, had a known *NF1* pathogenic variant, and a significant burden of cNFs.

### Cutaneous Neurofibroma measurement protocol

**Fig. 1:**
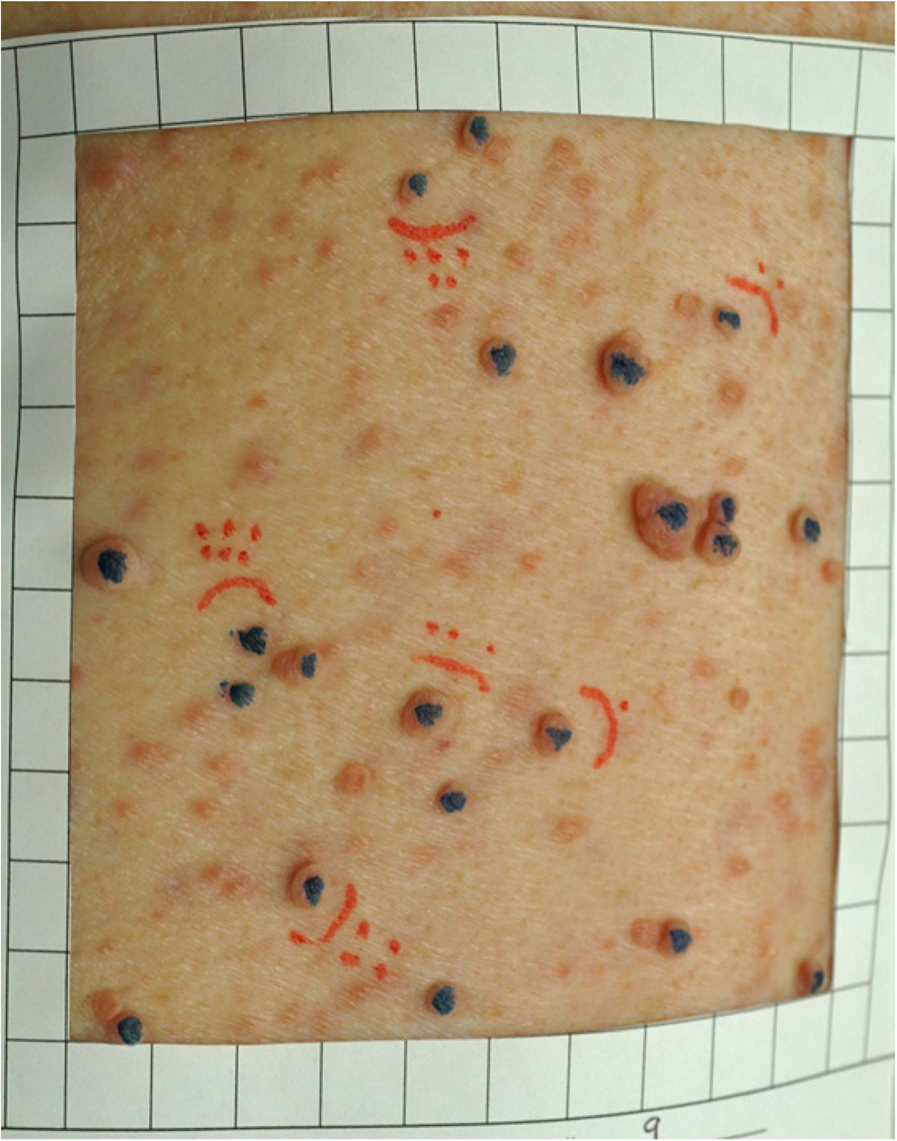
Picture of the 100cm^2^ paper frame used to count and measure cutaneous neurofibromas. Blue indicates cNFs that were counted (> 4 mm). Red indicates the cNFs that were measured

Paper frames with a 100cm^2^ cut-out area were used to guide the assessment (Fig. 1). The frames were made from paper with a sticky backing and easily adhered to skin. These frames were placed on 3 body sites: back, abdomen, and either the thigh or upper arm. Visible landmarks were used to align the frames consistently when measurements were made at different times, using photographs to insure similar positioning from one time to the next (the neurofibromas themselves can provide clear landmarks). A working definition was developed for operational purposes to reliably count and measure cNFs: dome-shaped, soft, fleshy bumps on the skin greater than 4 mm (calipers were used to determine the 4 mm minimum size threshold). Subcutaneous bumps that were firm and nodular were excluded because these are more difficult to accurately measure.

The cNF number measurement protocol was previously described in detail [15]. Briefly, all cNF counts were performed manually in “real time” by marking cNFs with a washable marker to avoid counting a tumor more than once, then photographed, and counted again by digital photography to confirm accuracy. All cNFs within the paper frame that fulfilled the working definition of a cNF were counted.

The cNF number measurement protocol was used as a guide to develop a method of measuring cNF volume. Instead of counting cNFs, digital calipers were used to manually measure the length, width, and height, which were used to calculate volume. The length measurement was made by placing the caliper’s outside jaws at the cNF’s outermost points with the longest diameter. The width measurement was made perpendicular to the length measurement. The height measurement was made by placing the terminus of caliper’s depth gauge at skin level and measuring to the topmost point of the cNF. Three to 6 target cNFs were identified within each paper frame, marked with washable marker, measured with a digital caliper, and photographed. Regions with plexiform neurofibromas, which involve multiple fascicles of the length of a nerve and its branches, may be large and disfiguring tumors, and usually develop in childhood, were not included within the measurement regions.

### Size measurement pilot study

A set of 16 plaster model “tumors” of known volume were utilized that ranged from 1.18–452.39mm^3^ in volume, 2-12 mm in diameter, and 1-6 mm in height. Three investigators (I1, I2, I3) measured the length, width, and height of the model tumors with calipers to calculate volume on 2 separate occasions.

### Prospective natural history study

A cohort of 22 adult patients with NF1 participated in the cNF natural history study. The cNF size and number protocol were performed at baseline, then every 4 months for 24 months (2 years). The measurement protocol was performed again approximately 96 months (8 years) after the baseline study visit. All 22 participants completed the study visits for 24 months and 14 were able to complete the 96 month study visit; the remaining 8 participants were unable to complete the final study visit because of loss to follow-up, death, or illness.

### Statistical analyses

To assure the reproducible and accurate measurement of cNF size in the pilot study, intraclass correlation coefficients (ICC) [16] were used to evaluate the agreement of measures from three investigators using laser scanner as well as the agreement between those measures and the actual model volume. High ICC (close to 1) among three investigators indicates high reproducibility and high ICC (close to 1) between human measures and actual volume indicate high accuracy of the measurements.

To account for any cNFs that were removed over the course of the study, investigators asked study participants at each visit if any cNFs were surgically removed. These cNFs were not included in the analyses. The size and number measurements obtained during the natural history study of cNF were analyzed separately. In order to provide average growth rates by region (back, abdomen, arm/leg), growth rate per cNF was estimated by change in volume. To account for clustering of lesions within a patient and the correlation from measuring multiple lesions per subject over time, random effect modeling was used with intercept and time as random effects and two level of clustering (i.e. lesions nested within patients). The time effects on the number of lesions were analyzed with a random effect model with the intercept and time as random effects and only one level of clustering (i.e. patients). The slope value shows the rate of increase by month. In both analyses, the Kenward-Roger approximation was used in standard error estimations and the F test, accounting for the small number of patients. All the analysis was conducted using SAS 9.4 (Cary, NC).

## Results

A pilot study was designed to determine if a caliper-based protocol for measuring cNF size was accurate and reliable. A set of 16 plaster model “tumors” of known, yet varying, volumes were utilized. Three investigators measured the length, width, and height of the model tumors with digital calipers to calculate volume on 2 separate occasions. Intraclass correlation coefficients (ICC) were derived from calculated volumes to assess 1) measurement accuracy to known model volume and 2) reproducibility for repeated measures (Table 1). The ICC showed that the investigators’ manual measurements were accurate (Table 1, last column) and reproducible (ICC = 0.9964). Therefore, the study investigators proceeded with using the caliper-based size measurement protocol for the duration of the cNF natural history study.

**Table 1:** Intraclass correlation of three investigators (I1, I2, I3) calculated volumes to actual model volumes (Actual Volume)

	I1	I2	I3	Actual Volume
I1	0.9958	0.9964	0.9964	0.9992
I2		0.9951	0.9964	0.9983
I3			0.9966	0.9936

This approach, as well as the previously designed cNF number measurement protocol, was employed to prospectively monitor tumor number and size in a cohort of 22 adult patients with NF1 over an 8-year period. The cNF size and number measurement protocol was performed at baseline, then every 4 months for 24 months (2 years), and finally after approximately 96 months (8 years) from the study initiation. All 22 participants completed the study visits for 24 months and 14 were able to complete the 96 month study visit; the remaining 8 participants were unable to complete the final study visit. Study participant descriptive information is detailed in Table 2. The mean age at baseline was 50.5 years and 54.5% were female.

**Table 2:** Study Participant Characteristics

Patient ID	Sex	Enrollment Age Range	Months Evaluated	NF1 Variant	Inheritance
1	F	60–69	96	c.4267A > G (p.K1423E)	Sporadic
2	F	60–69	96	c.60 + 1G > A	Sporadic
3	F	50–59	96	c.204 + 1G > A	Familial
4	M	50–59	96	c.5453delT	Familial
5	M	30–39	96	c.2693 T > C (p.L898P)	Familial
6	M	40–49	24	c.3113 + 1G > A	Sporadic
7	F	40–49	96	c.1527 + 1167C > G	Sporadic
8	F	30–39	24	c.3379delA	Familial
9	M	30–39	24	c.288 + 1G > T	Familial
13	F	40–49	24	c.2446C > T (p.R816X)	Familial
14	M	60–69	96	c.2041C > T (p.R681X)	Familial
15	F	40–49	24	c.2044C > T (p.Q682X)	Familial
16	M	70–79	24	c.1536dupA	Sporadic
18	M	40–49	96	c.3639_3641delAAT	Familial
19	M	50–59	96	c.7127-4insAlu	Familial
20	M	40–49	96	c.1890_1918dupT	Sporadic
21	F	30–39	96	c.6662delC	Familial
22	M	60–69	96	c.6138dupT	Familial
23	F	40–49	24	c.7519C > T (p.Q2507X)	Sporadic
24	F	60–69	24	c.7806 + 1G > A	Familial
25	F	30–39	96	c.7486C > T (p.R2496X)	Familial
26	F	60–69	96	c.7486C > T (p.R2496X)	Familial

**Fig. 2:**
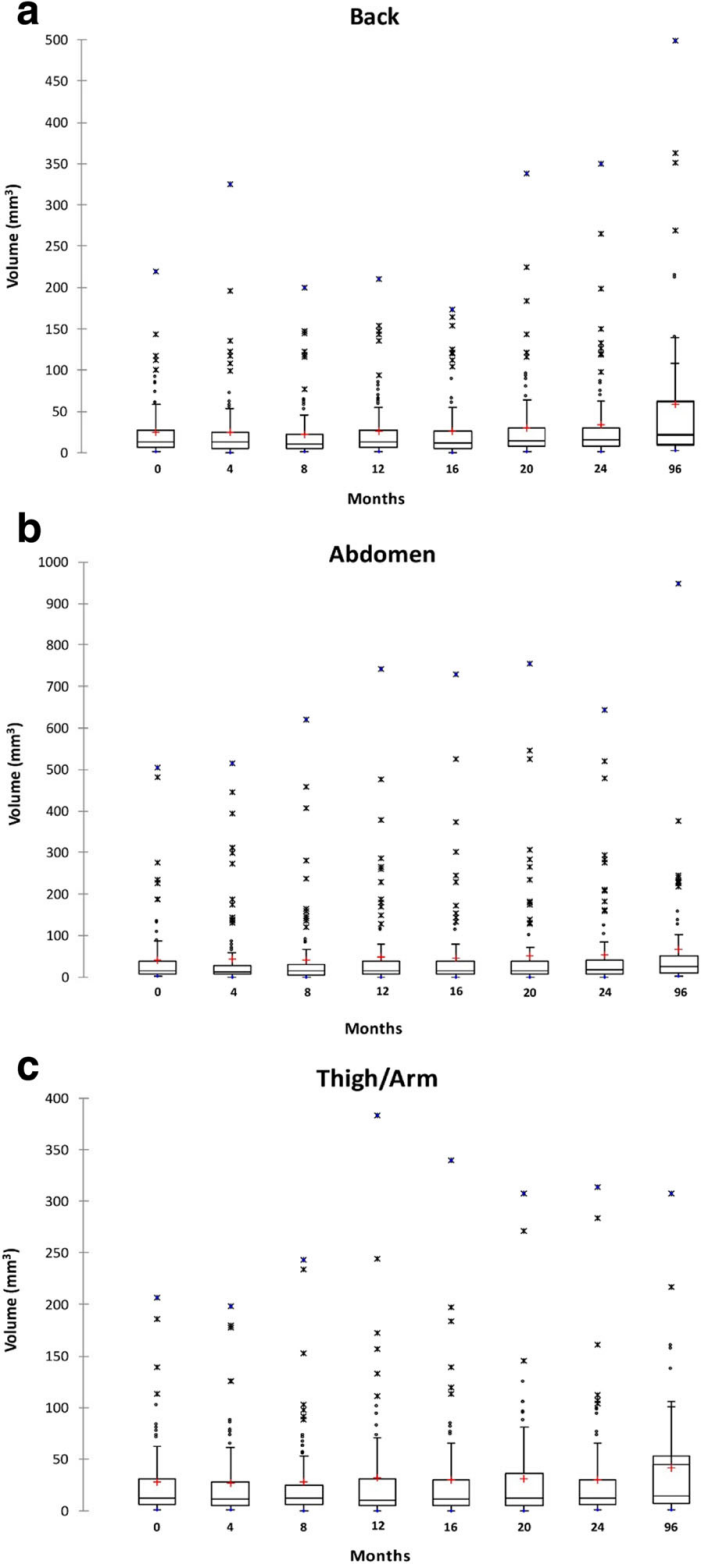
Cutaneous neurofibroma volume by body region over 96 months. Box-and-whisker plot shows the distribution of cutaneous neurofibroma volumes from the 22 patients over 96 months in the back region (**a**), abdominal region (**b**), and the upper arm or thigh (**c**). The circles (o) above the boxplot maximum range are suspected outliers, the stars (*) further above are outliers, the red plus (+) is the mean

To account for repeated non-independent size measurements of the same lesions per subject, random effect models were employed. Random effect modeling of the data from the 22 patients indicated that the average monthly increase in elliptical volume for cNFs was 0.37 mm^3^ in the back region (95% CI (0.23, 0.51), *p* < 0.0001; Fig. 2a), 0.28 mm^3^ in the abdominal region (95% CI (0.16, 0.41), p < 0.0001; Fig. 2b), and 0.21 mm^3^ in the arm/leg region (95% CI (0.08, 0.34), *p* = 0.0022; Fig. 2c). This means the average cNF volume increased by 2.6 fold in the back, 1.64 fold in the abdominal, and 1.75 fold in the leg/arm regions over 96 months.

**Fig. 3:**
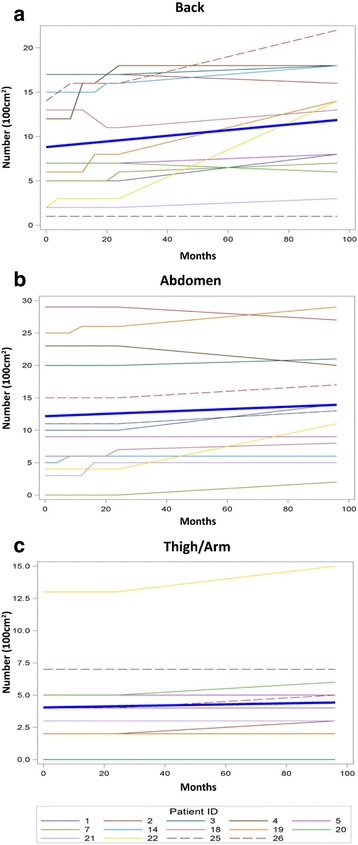
Cutaneous neurofibroma number by body region over 96 months. Line graph demonstrates the number of cutaneous neurofibromas in the back region (**a**), abdominal region (**b**), and the upper arm or thigh (**c**) for each study participant that completed all study visits. The thick blue line represents the slope

**Fig. 4:**
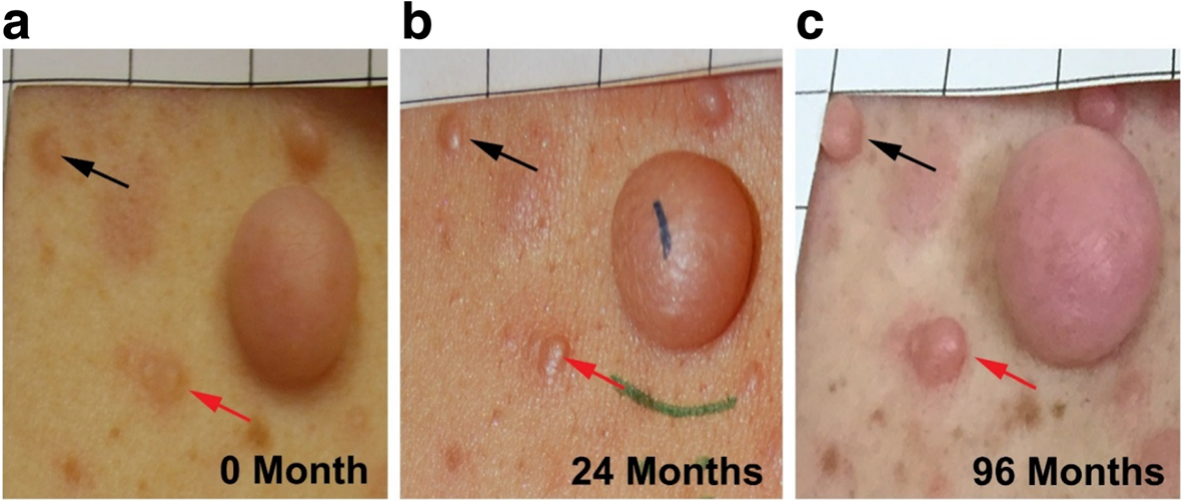
Visualization of cutaneous neurofibroma growth over 96 months. Black and red arrows denote 2 different cNFs at baseline (**a**), 24 months (**b**) and 96 months (**c**) that exhibited significant growth. Photos taken from the abdominal region of Patient 26

To evaluate longitudinal effects on the number of cNFs, random effect modeling was performed with both intercept and slope (time) as random effects. Random effect modeling from the 22 patients yielded a non-significant increase in cNF number during 96 months; however, in a subgroup analysis of the 14 patients that completed the 96 month study visit showed that the number of cNFs significantly increased in the back (slope = 0.032, *p* = 0.011) and abdominal (slope = 0.018, *p* = 0.026) regions, while the leg/arm regions retained a positive trend (slope = 0.004, *p* = 0.055) (Fig. 3). This means that the approximate cNF number increase was 3.1 in the back, 1.7 in the abdominal, and 0.4 in the leg/arm regions over the 8 year timespan. Interestingly, a study participant with a relatively large burden of cNFs had 2 new abdominal lesions develop in close proximity over the course of the study that was captured by photography (Fig. 4).

## Discussion

Quantitative analysis of a prospective natural history study of cNFs demonstrates that both size and number significantly increase over time, thereby validating patient-reported outcomes and cross-sectional studies [1, 3]. This quantitative approach was able to detect average rate of cNF increase as well as individual variabilities. These findings will facilitate research on cNF development and clinical trials targeting cNFs.

The pilot study showed that caliper-based size measurements are accurate and reliable, although it is recognized that measurement of plaster models may be easier than measurement of actual neurofibromas. Furthermore, this approach for the natural history study was able to detect not only significant changes, but also subtle changes in even short periods of time. Caliper-based measuring is relatively inexpensive and does not require the equipment, space, and lighting requirements for other measurement methodologies like 3D laser scanners or 3D photography. Therefore, this approach could be widely utilized, even by clinicians and researchers with limited resources. It is important to note that the measurement protocol does have limitations. Foremost, investigators performing the protocol must agree on what is a measurable cutaneous neurofibroma. This seems obvious to most individuals familiar with NF1 patients; however, some cNFs may fuse together or only partially break the skin surface and a consensus must be agreed upon before beginning the protocol. There is a small cost associated with the photography and calipers as well as a time commitment to fully complete the protocol (see details below). To facilitate the measurement protocol, the authors have developed an investigator guide for manual cNF measurement (Additional file 1), which includes helpful tips and cNF inclusion/exclusion criteria that includes pictures to help other investigators interested in this approach. The authors have shared the protocol, guide, and provided in-person training to investigators from other medical institutions. The approximate time required for training was 1 h. The amount of time required for investigators to complete the baseline measurement protocol with identification of frame placement was around 1 h; the follow-up measurement protocol time ranged from 30 to 45 min, depending on participant cNF burden.

cNF growth is highly variable between individuals, body site (back vs. abdomen vs. arm/leg), and even within the same body region. For example, Patient 26 had an abdominal cNF that grew 33.64 mm^3^ (190.3% of baseline) over 96 months while an adjacent cNF grew 1.62 mm^3^ (17.7% of baseline) over the same period. The growth rates of the 3 body sites investigated also differed, with the back region exhibiting greatest increase in both cNF size and number, followed by the abdominal region, and the leg and arm region had the least growth. Future studies are needed to determine whether specific constitutional *NF1* pathogenic variants, the type and number of somatic mutations, tumor microenvironment, vascularization, trauma, or other factors contribute to growth variability. A few studies have investigated cNF genetics. Thomas and colleagues (2010) analyzed 89 cNFs from 3 unrelated patients and found that 64% had a detectable and unique *NF1* somatic mutation [17]. Furthermore, several genes associated with mismatch repair and regulatory cell cycle (e.g. *TP53, CDKN2A,* and *RB1*) have been implicated as modifiers of NF1-related tumors [18, 19], suggesting genetic alterations aside from the *NF1* gene may influence cNF development.

Current treatment options for cNFs include surgical excision, laser removal, or electrodessication. All of these approaches can address a proportion of cNFs with good patient satisfaction; however, multiple treatment sessions may be necessary and these treatments do not remove all cNFs, result in scarring, and additional tumors will likely reappear over time. Currently, there are no effective systemic or topical treatments for cNFs. The development of several systemic therapies targeting plexiform neurofibromas [20, 21] makes this type of treatment a promising possibility, especially given the histologic similarities between cutaneous and plexiform neurofibromas. The average cNF growth rates from this natural history study can be utilized to improve the design and interpretation of clinical trials targeting cNFs. Most clinical trials targeting NF-related tumors are 24 cycles (approximately 2 years), which is a sufficient amount of time to detect a significant change in cNF size. Furthermore, the accurate and reliable measurement protocol is a viable option for cNF clinical trial outcome measures. Given the variable cNF growth rates by body site, it is recommended to measure cNFs from the same body sites of study participants to reduce variability in outcome measures. These findings could also be used by researchers considering clinical trials of NF1 patients targeting non-cNF tumor types, because cNF response can be easily used as a secondary outcome measure.

Some limitations and strengths should be noted. The major limitation of this study is the average age of the cohort at study initiation was 50.5 years. Previous studies have demonstrated rapid growth of cNFs during puberty and pregnancy [1–3]. This suggests that cNFs may have variable rates of growth during the lifespan. Therefore, the present study would not capture rate differences that may occur at younger ages and during periods of known rapid change like puberty and pregnancy, nor would it predict lifetime cNF burden. In addition, the number of patients is small, which may affect the precision of the estimates. The major strength of this study is that it is the first reported prospective natural history study of cNFs. Furthermore, the quantitative measurement protocol is reliable, accurate, and inexpensive.

## Conclusion

Cutaneous neurofibromas can be accurately and reliably measured over a prolonged period of time. The average monthly increase in cutaneous neurofibroma number and volume is significant; however, the rate of increase is variable by individual and body region. These findings may provide insight into cutaneous neurofibroma development and benefit researchers considering clinical trials targeting cutaneous neurofibromas.

## Additional file

Additional file 1: Investigator guide for manual cutaneous neurofibroma measurement. (PDF 354 kb)

## Abbreviations

cNFs: Cutaneous neurofibromas
ICC: Intraclass correlation coefficients
NF1: Neurofibromatosis type 1
