# Supplementary material for: Cutaneous neurofibromas in Neurofibromatosis type I: a quantitative natural history study
Source: Orphanet J Rare Dis. 2018 Feb 7;13:31. doi: 10.1186/s13023-018-0772-z (PMC5803843; doi:10.1186/s13023-018-0772-z)

INVESTIGATOR GUIDE FOR MANUAL CUTANEOUS NEUROFIBROMA MEASUREMENT

Supplies:

- Digital caliper
- Small caliper for minimum (4mm) count threshold
- Washable markers x2 colors
- 100cm^2 paper frames

Frame placement:

- Place 100cm^2 paper frames on 3 body sites for measurement outcomes: back, abdomen, and either the thigh or upper arm
- Frames should be on a flat or nearly flat surface that is easily accessible (e.g. body curvatures make photography difficult, inner thighs are difficult to measure)
- Try to use physical landmarks as a guide (e.g. cherry angioma)
- Frame must contain sufficient cNFs (at least 3 cNFs >4mm)

Photography:

- At baseline and study completion take whole body and regional pictures
- Take pictures of frames before and after measurements are made
- Photograph top surface, not at an angle

Volume Measuring Scheme:

- Identify 3-5 cNFs per frame for manual volume measurement
- Mark measured cNFs with a number using washable marker
- For the length measurement place the caliper's outside jaws at the cNF's outermost points with the longest diameter. For the width measurement place jaws perpendicular to the length measurement. For the height measurement place the terminus of caliper's depth gauge at skin level (do not compress skin) and measuring to the topmost point of the cNF
- 2 people are needed for efficient measurement (one will measure while the other records)

Counting Scheme:

- Count all cNFs ≥4mm within the frame (a small caliper set to 4mm is helpful)
- Mark cNF (use a different color than one used for measurements) as they are counted to prevent double counts

Inclusion/exclusion criteria:

- Exclude cNFs that are not fully within the frame

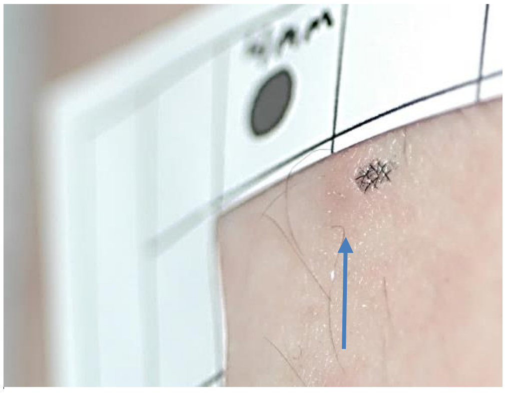

- Exclude satellite cNFs for measurement

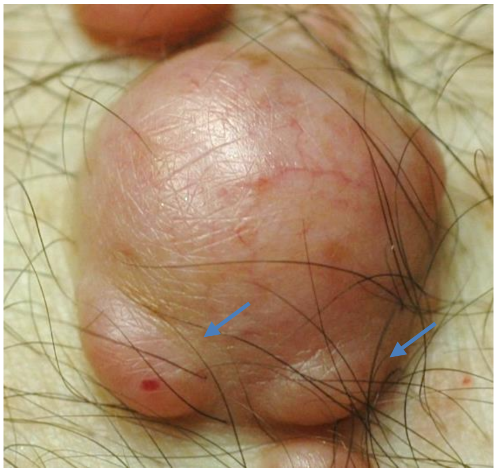

- Include adjacent cNFs with separate bases and count as 2 cNFs

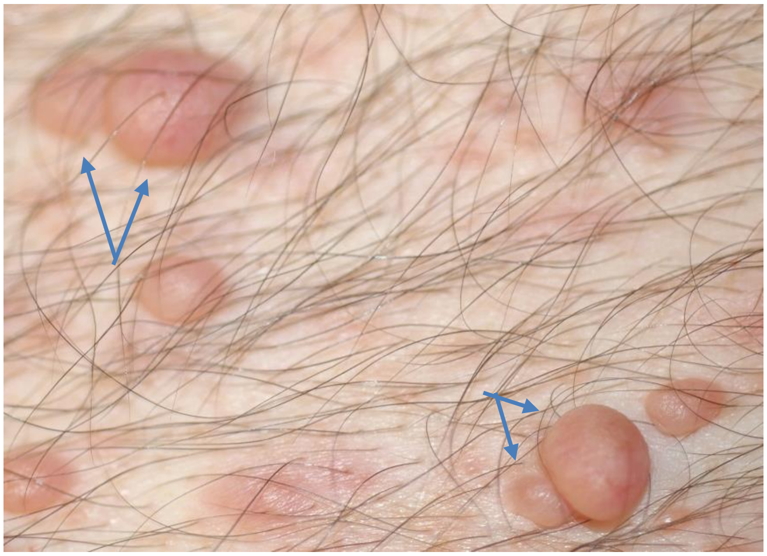

- Exclude fused cNFs fore measurement and count as 1 cNF

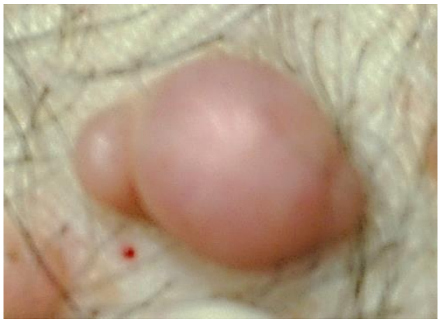

- Include deep cNFs for counts but **do not** measure

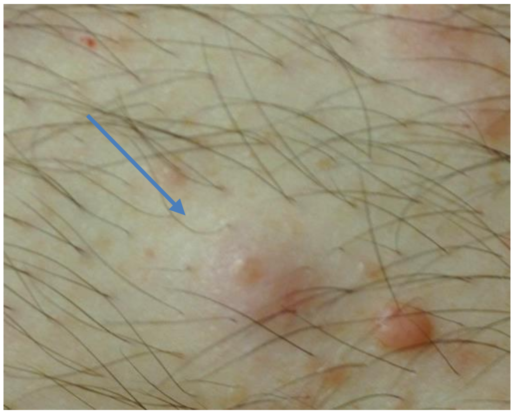

Supplement: Additional file 1: — Investigator guide for manual cutaneous neurofibroma measurement. (PDF 354 kb) [file 13023_2018_772_MOESM1_ESM.pdf]
